# Factors related to dietary quality among older stroke high-risk population in Tianjin community, China: a multicenter study

**DOI:** 10.1186/s12877-023-04211-7

**Published:** 2023-08-22

**Authors:** Yumeng Gu, Decheng Gao, Xiaoshuang Xia, Juanjuan Xue, Dongliang Wang, Zhiqiang Wei, Xiaolin Tian, Xin Li

**Affiliations:** 1 Department of Neurology, Second Hospital of Tianjin Medical University, No.23, Pingjiang Road, Hexi District, Tianjin, 300211 China; 2 Department of Rehabilitation, Second Hospital of Tianjin Medical University, Tianjin, 300211 China

**Keywords:** Stroke risks, Diet Quality, AHEI-2010, Tianjin Community

## Abstract

**Background:**

Stroke is a common and frequently-occurring disease in older people. It has the characteristics of high morbidity, high mortality, high recurrence rate and high disability rate. Most stroke risk studies are based on pathophysiology, however psychosocial factors such as diet quality are often understudied. The aim of this study was to assess stroke risk in urban community residents in Tianjin and investigate the factors that affect the dietary quality of older stroke high-risk populations.

**Methods:**

Using a cross-sectional, multicenter study, recruit people aged 60 to 80 in Tianjin. Dietary intake data were obtained through a validated food frequency questionnaire, which were used to calculate Alternate Healthy Eating Index-2010 (AHEI-2010) and to analyze its association with sociodemographic characteristics, stroke risk factors and health marker variables.

**Results:**

A total of 1068 participants from 4 community health service centers in Tianjin were recruited, including 300 low-risk individuals and 768 high-risk individuals. Compared with the low-risk group (62.75 ± 3.59), the AHEI-2010 mean score of the high-risk group (56.83 ± 6.54) was significantly lower. The top three most common risk factors among participants were dyslipidemia (80.3%), hypertension (60.6%), and physical inactivity (58.2%). Multiple logistic regression showed that diet quality was independently and significantly associated with stroke risk (OR = 0.765; 95%CI: 0.690–0.848, *p* < 0.001).

**Conclusion:**

The diet quality of high-risk stroke population in Tianjin is far from ideal. At the same time, public health knowledge needs to be disseminated and educated, especially among those at high risk of cerebrovascular disease, with a focus on improving psychosocial factors such as diet quality.

**Supplementary Information:**

The online version contains supplementary material available at 10.1186/s12877-023-04211-7.

## Background

Stroke is a common cause of death in the Chinese population [1], and has surpassed heart disease as the leading cause of death and adult disability [2]. The prevalence and incidence of stroke have risen faster than in other countries [3]. In addition, the burden of stroke in China is also increasing [4].

The major risk factors for stroke are hypertension, diabetes, smoking, and hyperlipidemia, as well as lifestyle factors such as obesity, diet/malnutrition, and reduced physical activity [5]. Most of the causes of disease and death worldwide are closely related to dietary factors, and the application of dietary indices has been shown to be a useful tool for determining the quality of a population’s diet [6]. Dietary factors are one of the major contributors to the burden of disease and death in both developed and developing countries. Dietary factors are closely related to chronic non communicable diseases, including cardiovascular disease, type 2 diabetes and some types of cancer [7].

Using statistical methods or scoring indices to quantify adherence to a given dietary pattern, the relationship between diet quality (DQ) and health can be examined [8]. The Alternative Healthy Eating Index (AHEI) was created in 2002 based on foods and nutrients that predict chronic disease risk. It was used to assess adherence to the Harvard Healthy Eating Plate. Higher AHEI scores were associated with lower risk of major chronic diseases (cardiovascular disease, diabetes, colorectal and breast cancer) and lower overall mortality [9]. AHEI-2010 has been widely used in China to evaluate the dietary quality of different diseases and populations [10–14]. In this study, we attempted to explore specific dietary patterns and behaviors associated with stroke risk. Conduct a detailed analysis of each component of AHEI-2010 to explore the specific food categories that have led to changes in the dietary quality of the older stroke high-risk population in Tianjin. Provide key points and basis for promoting and educating the dietary habits of high-risk older stroke populations in the next step.

## Methods

### Study design

This study is part of the China’s National Stroke Screening and Prevention Program (CSPP) [15–19], a community-based cross-sectional study managed by the National Stroke Prevention and Control Program Office and conducted in 31 provinces in Chinese Mainland. Using a two-stage stratified cluster sampling method. At the first stage, 200 project areas are first determined based on the proportion of local population size and total county size. At the second stage, based on the geographical environment and suggestions from local hospitals, one urban community and one rural area are selected as the main sampling units in each project area. In accordance with the instructions of the National Health and Family Planning Commission and the Tianjin Municipal Health and Family Planning Commission, our unit was responsible for conducting stroke screening in four communities that matched the sixth national census and were similar to the population distribution of Tianjin. By cluster sampling, all residents aged 40 or above in the selected community are invited to participate in the screening plan, which is carried out in the nearby community hospital or health station. Questionnaire survey, physical examination and stroke risk factors assessment were conducted in primary health care institutions. Based on this task, this study added food frequency questionnaires (FFQ) to the 60–80 year-old participants. The study was approved by the Ethics Review Committee of the Second Hospital of Tianjin Medical University.

The survey includes a health interview, a health check and a nutritional assessment. Family doctor by phone or during a clinical visit. If the participant is interested in participating, a face-to-face interview was arranged to explain the purpose and characteristics of the study [20, 21]. During the baseline phase of the initial study, sociodemographic information (e.g., age, socioeconomic status, education), medical history, anthropometric measurements (weight, height, and waist circumference), diet, physical activity, and blood pressure were recorded, and blood samples were collected. All participants signed informed consent.

Exclusion Criteria: Individuals under 18 years of age, pregnant and breastfeeding women, individuals with severe physical or mental impairment affecting food intake and physical activity.

**Fig. 1:**
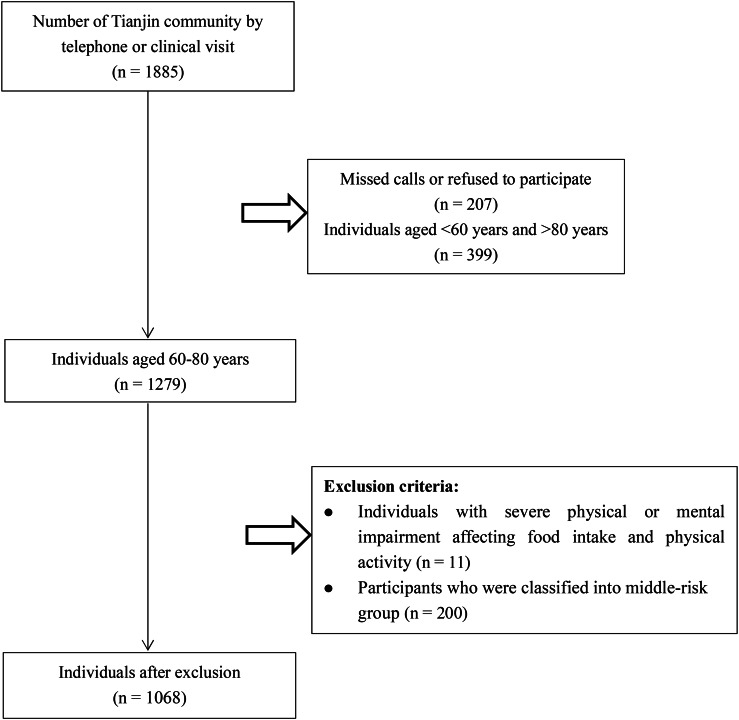
Flowchart of Tianjin Community Population Participation Study

For this study, we recruited 60–80 year old in the Tianjin community from October 2020 to December 2021, and performed the final analysis on data from 1068 adults (Fig. 1).

### Dietary assessment

Diet was measured by a modified 1995 version of the diet frequency questionnaire (FFQ), which had been previously validated against dietary records [22, 23]. FFQ has undergone multiple verifications in China and has achieved reasonable validity and reproducibility [24–27]. The FFQ includes 103 foods (20 beverages and 83 solid foods), which account for more than 90% of the intake of most nutrients [28].

Trained personnel took a quick ration of each food or drink, prescribed a common serving size (such as 1/2 cup of carrots or 2 slices of bacon), and asked participants how much of this amount they consumed on average in the previous year frequency [29]. Up to nine predefined intake frequencies, ranging from never to ≥ 5 times/day, each drink has three predefined serving sizes, from small to large. For solid foods, the predefined frequency ranges from never to ≥ 2 times/d. Total energy intake and nutrient intake were calculated by multiplying the reported frequency, the reported portion size and the corresponding nutrient content [30]. If the dietary information on the form is incomplete, the questionnaire will be considered invalid. The validity and reliability of this FFQ is well documented with respect to nutrients and food consumption [31].

### AHEI-2010 application

AHEI-2010 is an alternative measure developed by researchers at the Harvard School of Public Health. The method is a commonly used diet quality measurement tool and is calculated using data from the FFQ [32, 33]. Dietary studies tend to focus on the overall diet level rather than a single food group because dietary components are eaten together and interact. AHEI-2010 is based on evidence-based medical recommendations. This recommendation provides dietary advice that better improves health risk factors and is more closely associated with reducing chronic disease risk [9, 34]. Therefore, we believe that stroke risk may be offset by changes in lifestyle and diet. In clinical and epidemiological investigations, we sought to capture specific dietary patterns and eating behaviors associated with lower stroke risk. AHEI-2010 consists of 11 components, including 6 components used to assess adequacy (vegetables, fruits, whole grains, nuts and legumes, long-chain (ω-3) fats and PUFA) and 5 components used to assess moderation (sugary beverages and juices, red/processed meat, trans fat, sodium and alcohol). The AHEI-2010 total score is calculated as the sum of the individual scores for the 11 components. All AHEI-2010 sub-scores range from 0 to 10. Meanwhile, the AHEI-2010 total score ranges from 0 (non-adherence) to 110 (best compliance with a healthy diet) [9, 32]. The consumption amount between the lowest and highest possible scores will be scored proportionally based on its consumption amount. All components except sodium are scored based on absolute values. Sodium scores are derived from the deciles of sodium consumption in the sample population. Details of calculating AHEI-2010 have been published elsewhere [9].

### Sociodemographic assessment

Health interviews were used to understand the demographic and socioeconomic characteristics of the participants, including age, health status, education level, marital status, income, smoking status, alcohol consumption, and physical activity.

Health conditions include doctor-diagnosed chronic diseases. The education level includes the following three categories: below high school, high school and above. There are two types of marital status: married and single. Income is divided into three levels: below 10 thousand, 10 to 20 thousand, and above 20 thousand.

Blood samples were taken to measure health markers: fasting blood glucose (FBG), cholesterol, serum triglycerides (TG), serum high-density lipoprotein (HDL) cholesterol, low-density lipoprotein cholesterol (LDL), and homocysteine (HCY).

### Stroke risk factors

Stroke risk stratification based on the National Stroke Association’s Stroke Risk Scorecard, which collects 8 risk factors for stroke: high blood pressure, diabetes, dyslipidemia, atrial fibrillation (AF), current smoking, overweight or obesity, physical inactivity, and family history of stroke [17, 35].

According to the Chinese National Stroke Screening Survey (CNSSS) [15], the definitions for each risk factor are as follows: hypertension was the mean of two resting blood pressure measurements, systolic blood pressure ≥ 140 mmHg or diastolic blood pressure ≥ 90 mmHg, or participant reported a history of hypertension or use of antihypertensive medication [36]. Diabetes refers to use of antidiabetic drugs, or self-reported history of diabetes or FBG ≥ 7.0 mmol/L [37]. Dyslipidemia refers to the use of lipid-lowering drugs or at least one of the following: TG ≥ 1.70 mmol/L, cholesterol ≥ 5.18 mmol/L, low-density lipoprotein cholesterol ≥ 3.37 mmol /L [38]. AF was defined as self-report or on-site diagnosis by ECG. Overweight or obesity is defined as a BMI ≥ 28 kg/m^2^ [39]. Current smoking means smoking ≤ 1 cigarette per day. Physical inactivity is defined as less than 3 times a week of physical activity lasting less than 30 min, including industrial and agricultural labor. Family history of stroke was limited to immediate family members. Participants with ≥ 3 above-mentioned risk factors were classified into high-risk group. Participants with < 3 of the above risk factors without any of the three risk factors for hypertension, diabetes, and AF were classified as low risk.

### Statistical analysis

Diet quality in high-risk stroke populations was divided into five groupsaccording to AHEI-2010 using 5 quantile [40–42]. Use the Kolmogorov-Smirnov test to determine whether the data distribution is normal, continuous variables were expressed as mean ± standard deviation or median (interquantile range). Categorical variables are expressed as counts and proportions.Analyses were performed using independent samples t-test, one-way ANOVA, or Mann-Whitney U test.

Differences between groups were assessed using Bonferroni correction for multiple comparisons. Logistic regression analysis of the association between dependent and covariates. All analyses were performed using SPSS Statistics version.

## Results

A total of 1068 participants in this study completed the questionnaire, which included 300 low-risk participants and 768 high-risk participants. The mean age of the participants was 69.22 ± 11.21, ranging from 60 to 80 years old. More women than men (55.5% vs. 44.5%). Married participants had the most (90.0%). High school education or above accounted for the largest proportion (68.4%).

### Stroke risk

Table 1 shows the prevalence of each of the eight risk factors, the most common risk factor was dyslipidemia (80.3%), followed by hypertension (60.6%) and physical inactivity (58.2%). The least common risk factor was atrial fibrillation, which was reported by only 70 participants (6.60%). Combining the eight risk factors, there were 300 (28.1%) in the low-risk stroke group and 768 (71.9%) in the high-risk group.

**Table 1:** Eight risk factors for stroke

Characteristics		n (%)
Risk factors for stroke		
Hypertension	No	421 (39.4)
	Yes	647 (60.6)
Dyslipidemia	No	210 (19.7)
	Yes	858 (80.3)
Diabetes mellitus	No	794 (74.3)
	Yes	274 (25.7)
Atrial fibrillation	No	998 (93.4)
	Yes	70 (6.60)
Current smoking	No	855 (76.9)
	Yes	213 (19.9)
Over weight or obesity	No	821 (76.9)
	Yes	247 (23.1)
Sufficient physical activity	No	446 (41.8)
	Yes	622 (58.2)
Family history of stroke	No	618 (57.9)
	Yes	450 (42.1)
Stroke risk level	Low risk	300 (28.1)
	High risk	768 (71.9)

### Comparison between the two stroke risk groups

Table 2 shows a comparison of sociodemographic characteristics, stroke risk factors, diet quality, and health indicators between low and high-risk stroke populations.

**Table 2:** Baseline demographics and clinical characteristics of all participants

Variables	Low risk (n = 300)	High risk (n = 768)	*p*-value
Age, years	66.63 (9.15)	66.09 (9.02)	0.381
Female, n (%)	169 (56.3)	424 (55.2)	0.740
Educaton Level			< 0.001
Below high school	13 (4.3)	324 (42.2)	
High School and above	287 (95.7)	444 (57.8)	
Marital Status			0.488
Current Married	273 (91.0)	688 (89.6)	
Single	27 (9.0)	80 (10.4)	
Annual household income (¥)			0.629
< 10,000	23 (7.7)	59 (7.7)	
10,000–19,999	31 (10.3)	65 (8.5)	
≥ 20,000	246 (82.0)	622 (83.9)	
Current drinking, n (%)	52 (17.3)	146 (19.0)	0.526
Stroke related risk factors			
Hypertension	0	647 (84.2)	< 0.001
Dyslipidemia	210 (70.0)	648 (84.4)	< 0.001
Diabetes mellitus	0	274 (35.7)	< 0.001
Atrial fibrillation	0	70 (9.1)	< 0.001
Current smoking	52 (17.3)	161 (21.0)	0.182
Over weight or obesity	47 (15.7)	200 (26.0)	< 0.001
Sufficient physical activity	140 (46.7)	482 (62.8)	< 0.001
Family history of stroke	93 (31.0)	357 (46.5)	< 0.001
AHEI−2010			
Total	62.75 (3.59)	56.83 (6.54)	< 0.001
Vegetables	7.21 (1.81)	6.31 (1.14)	< 0.001
Fruit	2.08 (0.49)	1.72 (0.45)	< 0.001
Whole grains	6.09 (1.40)	6.17 (1.34)	0.387
Sugar-sweetened beverages and fruit juice	5.40 (0.74)	5.58 (0.65)	< 0.001
Nuts and legumes	5.99 (0.51)	4.98 (0.59)	< 0.001
Red/processed meat	5.13 (0.69)	4.42 (0.67)	< 0.001
trans Fat	7.27 (0.84)	7.24 (0.82)	0.594
Long-chain (ω-3) fats (EPA + DHA)	5.49 (0.70)	4.52 (0.78)	< 0.001
Polyunsaturated fatty acids	7.38 (0.81)	7.01 (1.41)	< 0.001
Sodium	7.01 (1.18)	5.85 (0.83)	< 0.001
Alcohol	3.81 (0.53)	3.19 (0.57)	< 0.001
Health Marker			
BMI (kg/m^2^)	25.43 (23.17, 27.18)	25.61 (23.43, 28.21)	0.056
WC (cm)	90 (82, 97)	90 (82.5, 97)	0.638
Total cholesterol (mg/dl)	4.86 (3.95, 5.75)	4.99 (4.22, 5.85)	0.015
HDL cholesterol (mg/dl)	1.17 (1.01, 1.47)	1.21 (1.02, 1.46)	0.112
LDL cholesterol (mg/dl)	2.91 (2.22, 3.76)	3.07 (2.33, 3.78)	0.275
TG (mg/dl)	1.53 (1.10, 1.95)	1.53 (1.10, 2.25)	0.107
FBS (mmol/L)	5.2 (4.7, 5.93)	5.2 (4.7, 6.4)	0.054
Glycated hemoglobin (%)	5.05 (4.7, 5.8)	5.10 (4.7, 5.8)	0.660
Homocysteine (µmol/L)	13.7 (10.58, 18.2)	14.7 (11.2, 19.2)	0.095
Mean SBP (mmHg)	130 (120, 142)	132 (120, 142)	0.389
Mean VBP (mmHg)	80 (70, 86)	80 (70, 87)	0.770

Data are presented as mean ± standard deviation for normally distributed variables and percent for categorical variables

EPA + DHA: eicosapentaenoic and docosahexaenoic acids

In terms of sociodemographic characteristics, compared with the low-risk group, the high-risk group had a significantly lower level of high school education or above (*p* < 0.001). In terms of stroke risk factors, compared with the low-risk group, the high-risk group had higher prevalence of hypertension, dyslipidemia, diabetes, atrial fibrillation, overweight/obesity, and family history of stroke, with significant differences between the two groups. In terms of diet quality, compared with the low-risk group, the AHEI-2010 total score of the high-risk group was significantly lower, and the individual scores of vegetables, fruit, nuts and legumes, red/processed meat, long-chain (omega-3) fats (EPA + DHA), polyunsaturated fatty acids, sodium and alcohol were significantly lower. However, the high-risk group had significantly higher individual scores for sugar-sweetened beverages and fruit juice compared with the low-risk group. In terms of health indicators, total cholesterol in the high-risk group was significantly higher than that in the low-risk group.

### Comparison between AHEI-2010 quantile groups of high-risk groups

The characteristics of the high-risk group are shown in Table 3 of the quantiles of the AHEI-2010. Participants in the highest quantile of AHEI-2010 scores were more likely to be college or higher, married, and tended to have lower BMI, total cholesterol, TG, and FBS levels, and tended to have higher HDL levels. In addition, the lowest quantile was significantly lower in all components compared to the highest quantile, except whole grains. The above results indicate thatparticipants in the highest quantile of AHEI-2010 scores had significantly higher intakes of vegetables, fruits, nuts, EPA + DHA and PUFA (*p* < 0.05). Meanwhile, the intake of red and processed meat, sugar-sweetened beverages, trans fatty acids, salt, and alcohol consumption were more moderate (*p* < 0.05).

**Table 3:** Characteristics of high-risk population for stroke by quintile of Alternative Healthy Eating Index−2010 (AHEI−2010) scores

Variables	Q1 (n = 153)	Q2 (n = 154)	Q3 (n = 153)	Q4 (n = 154)	Q5 (n = 154)	*p*-value
Age, years	65.43 (9.50)	65.48 (8.57)	66.28 (9.01)	65.68 (9.24)	67.54 (8.73)	0.208
Female, n (%)	87 (56.9)	77 (50.0)	91 (59.5)	81 (52.6)	88 (57.1)	0.456
Educaton Level						< 0.001^b − g,i^
Below high school	65 (42.5)	52 (33.8)	94 (61.4)	109 (70.8)	124 (80.5)	
High School and above	88 (57.5)	102 (66.2)	59 (38.6)	45 (29.2)	30 (19.5)	
Marital Status						< 0.001^b,d^
Current Married	124 (81.0)	140 (90.9)	145 (94.8)	137 (89.0)	142 (92.2)	
Single	29 (19.0)	14 (9.1)	8 (5.2)	17 (11.0)	12 (7.8)	
Annual household income (¥)						0.109
< 10,000	9 (5.9)	13 (8.4)	11 (7.2)	18 (11.7)	8 (5.2)	
10,000–19,999	9 (5.9)	10 (6.5)	14 (9.2)	11 (7.1)	21 (13.6)	
≥ 20,000	135 (88.2)	131 (85.1)	128 (83.7)	125 (81.2)	125 (81.2)	
Current drinking, n (%)	34 (22.2)	22 (14.3)	34 (22.2)	27 (17.5)	29 (18.8)	0.342
Stroke related risk factors						
Hypertension	126 (82.4)	127 (82.5)	125 (81.7)	135 (87.7)	134 (87.0)	0.438
Dyslipidemia	129 (84.3)	125 (81.2)	130 (85.0)	132 (85.7)	132 (85.7)	0.798
Diabetes mellitus	53 (34.6)	57 (37.0)	63 (41.2)	58 (37.7)	43 (27.9)	0.164
Atrial fibrillation	19 (12.4)	10 (6.5)	11 (7.2)	13 (8.4)	17 (11.0)	0.314
Current smoking	29 (19.0)	37 (24.0)	31 (20.3)	33 (21.4)	31 (20.1)	0.849
Over weight or obesity	47 (30.7)	39 (25.3)	35 (22.9)	35 (22.7)	44 (28.6)	0.411
Sufficient physical activity	95 (62.1)	104 (67.5)	92 (60.1)	100 (64.9)	91 (59.1)	0.528
Family history of stroke	74 (48.4)	72 (46.8)	77 (50.3)	67 (43.5)	67 (43.5)	0.694
AHEI−2010						
Total	40.45 (3.57)	46.46 (1.20)	49.69 (0.88)	53.11 (1.01)	58.45 (2.99)	< 0.001^a − j^
Vegetables	5.08 (2.14)	5.87 (1.98)	6.19 (2.14)	6.80 (2.03)	7.61 (1.50)	< 0.001^a − d,f,g,i,j^
Fruit	1.34 (0.17)	1.46 (0.18)	1.62 (0.25)	1.85 (0.35)	2.32 (0.56)	< 0.001^a − j^
Whole grains	6.16 (1.33)	6.14 (1.28)	6.36 (1.28)	6.11 (1.37)	6.10 (1.42)	0.420
Sugar-sweetened beverages and fruit juice	5.74 (0.46)	5.71 (0.53)	5.63 (0.61)	5.52 (0.67)	5.30 (0.81)	< 0.001^c,d,g,i,j^
Nuts and legumes	3.21 (0.57)	4.51 (2.10)	5.19 (2.04)	5.56 (2.02)	6.39 (1.87)	< 0.001^a − g,i,j^
Red/processed meat	3.37 (0.20)	3.89 (0.97)	4.46 (0.93)	4.72 (0.72)	5.66 (1.38)	< 0.001^a − g,i,j^
trans Fat	7.18 (0.97)	7.28 (0.54)	7.20 (0.79)	7.37 (0.74)	7.15 (0.99)	0.126
Long-chain (ω-3) fats (EPA + DHA)^1^	2.58 (0.65)	4.49 (1.00)	4.50 (0.14)	5.29 (0.56)	5.69 (0.70)	< 0.001^a − d,f−j^
Polyunsaturated fatty acids	6.20 (2.22)	6.85 (1.33)	7.22 (1.06)	7.31 (0.90)	7.45 (0.64)	< 0.001^a − d,f,g^
Sodium	4.41 (0.76)	5.01 (0.59)	5.73 (0.44)	6.65 (0.32)	7.41 (1.95)	< 0.001^b − j^
Alcohol	2.60 (0.15)	2.60 (0.25)	2.91 (0.61)	3.34 (0.93)	4.53 (0.58)	< 0.001^b − j^
Health Marker						
BMI^2^ (kg/m^2^)	26.30 (24.22, 29.04)	25.39 (23.39, 28.57)	25.65 (22.97, 28.30)	25.71 (23.18, 27.54)	24.96 (22.97, 27.02)	0.003^c,d^
WC^3^ (cm)	90 (84, 97.25)	90 (83, 96)	89 (82, 97)	90 (82, 97)	90 (84.25, 97)	0.456
Total cholesterol (mg/dl)	5.08 (4.19, 5.74)	4.97 (4.34, 5.95)	5.34 (4.29, 5.90)	4.84 (3.87, 5.58)	4.83 (4.14, 5.73)	0.002^ h,i^
HDL^4^ cholesterol (mg/dl)	1.21 (1.00, 1.40)	1.23 (1.03, 1.47)	1.22 (1.08, 1.49)	1.18 (1.00, 1.48)	1.16 (0.99, 1.47)	0.030^i^
LDL^5^ cholesterol (mg/dl)	2.99 (2.26, 3.73)	3.15 (2.49, 3.78)	3.36 (2.47, 3.95)	2.82 (2.09, 3.75)	2.98 (2.28, 3.66)	0.111
TG^6^ (mg/dl)	1.74 (1.24, 2.56)	1.50 (1.06, 2.49)	1.53 (1.04, 2.38)	1.57 (1.11, 2.00)	1.46 (1.03, 1.82)	0.013^d^
FBS^7^ (mmol/L)	5.70 (4.90, 7.20)	5.20 (4.63, 6.90)	5.28 (4.60, 6.80)	5.30 (4.70, 6.10)	5.10 (4.50, 5.80)	0.002^c,d^
Glycated hemoglobin (%)	5.10 (4.70, 5.80)	5.10 (4.70, 5.80)	5.10 (4.70, 5.60)	5.20 (4.73, 5.90)	4.95 (4.53, 5.68)	0.325
Homocysteine (µmol/L)	15.45 (11.88, 19.30)	15.75 (11.63, 20.40)	13.70 (11.00, 19.00)	13.80 (11.05, 18.35)	13.65 (10.43, 18.18)	0.052
Mean SBP^8^ (mmHg)	135.50 (120, 145.25)	134 (123.25, 145)	132 (121, 142)	132 (122.25, 142)	130 (120, 142)	0.797
Mean VBP^9^ (mmHg)	80 (70, 88.25)	80 (70, 90)	80 (70, 85)	80 (70, 86)	80 (70, 87)	0.399

^a−j^ Mean Significant differences in correction for multiple comparisons, a: Q1 vs. Q2, b: Q1 vs. Q3, c: Q1 vs. Q4, d: Q1 vs. Q5, e: Q2 vs. Q3, f: Q2 vs. Q4, g: Q2 vs. Q5, h: Q3 vs. Q4, i: Q3 vs. Q5, j: Q4 vs. Q5; ^1^EPA + DHA: Eicosapentaenoic Acid + Docosahexaenoic Acid; ^2^BMI: Body Mass Index; ^3^WC: waist circumference; ^4^HDL: high-density lipoprotein; ^5^LDL: low-density lipoprotein; ^6^TG: Triglyceride; ^7^FBS: Fasting Blood Sugar; ^8^SBP: systolic blood pressure; ^9^VBP: venous blood pressure

### The influence of education and marital status on AHEI-2010 score

**Fig. 2:**
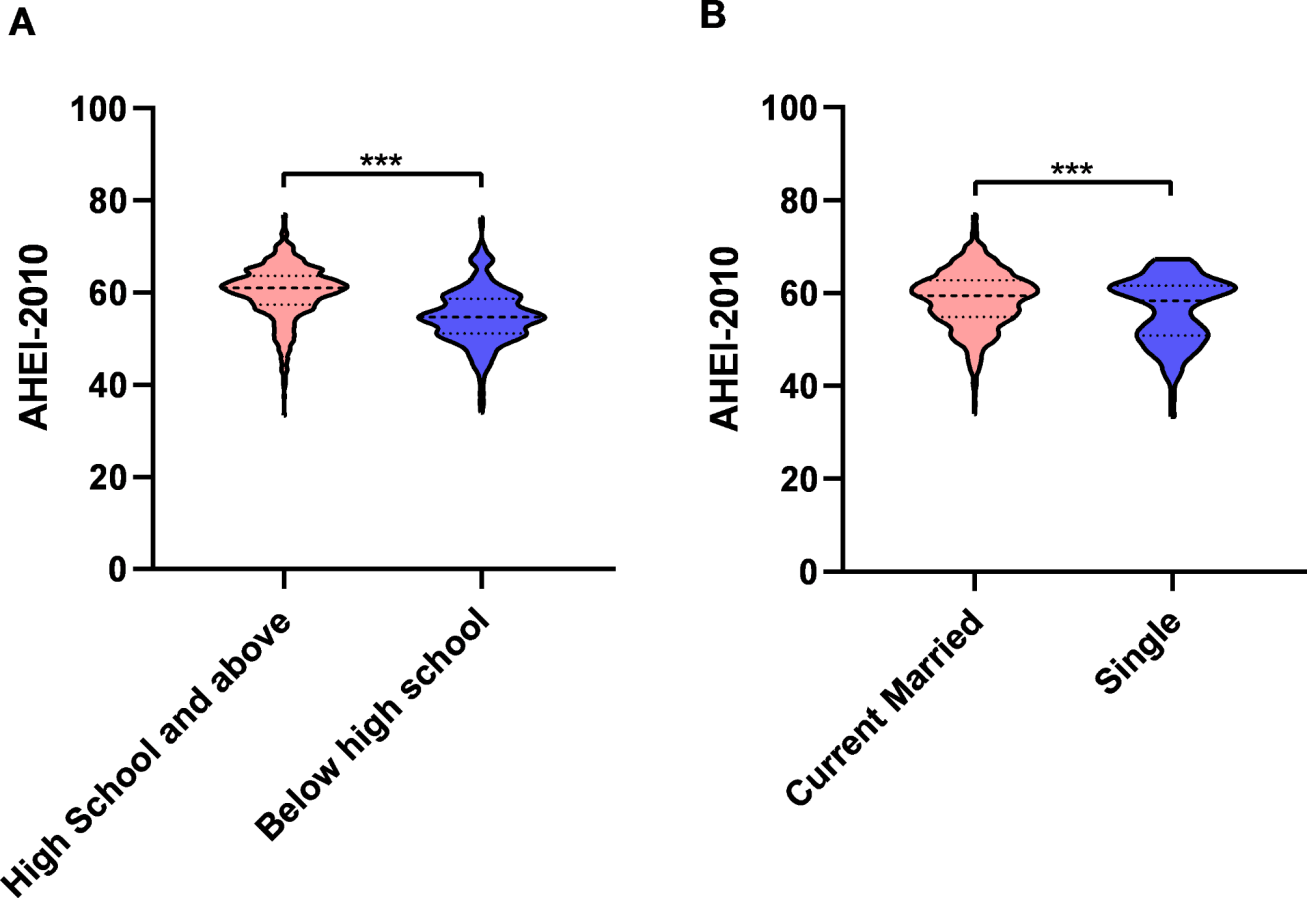
Total AHEI-2010 scores according to marital status and education level in Tianjin community, China. **(A)** Compared with below high school group, the AHEI-2010 score in high school and above group was significantly high (t-test, *p* < 0.001). **(B)** Compared with single group, the AHEI-2010 score in current married group was significantly high (t-test, *p* < 0.001). Error line representation SD. SD, Standard deviation. ***, *p* < 0.001

**Fig. 3:**
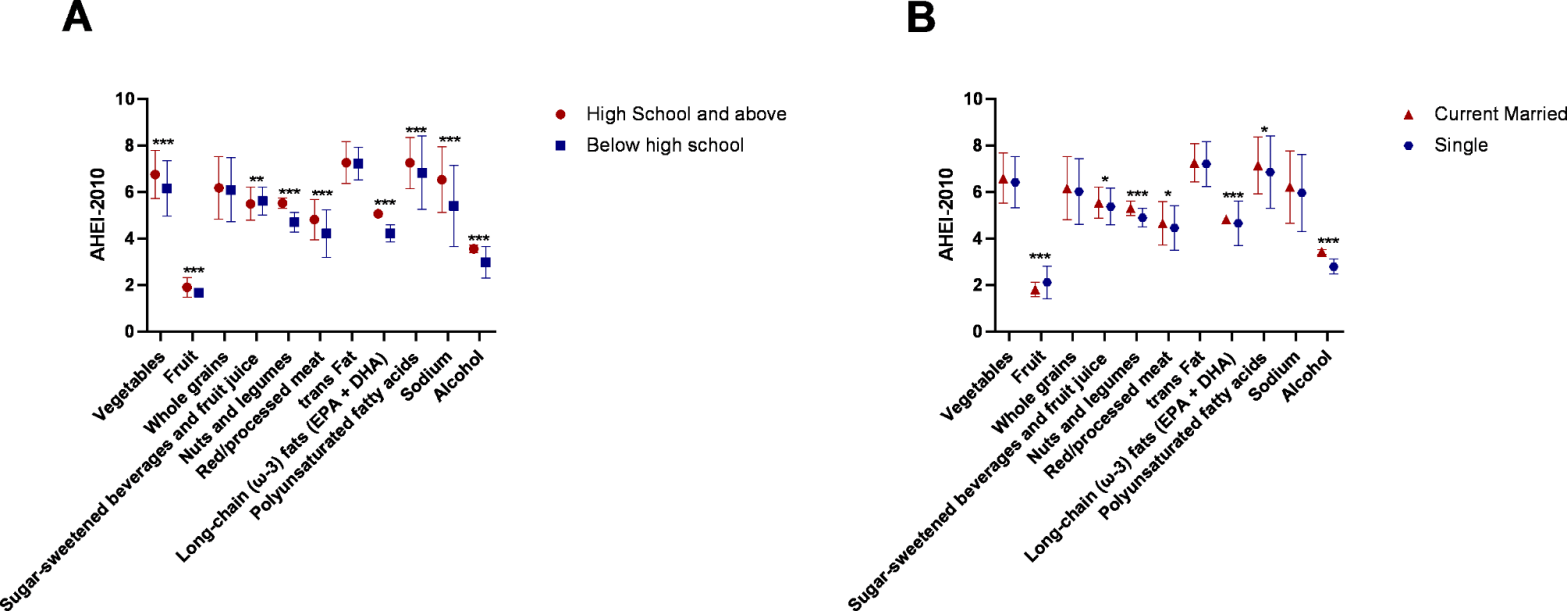
The AHEI-2010 single item score according to marital status and education level in Tianjin community, China. **(A)** Compared with below high school group, participants in high school and above scored higher in vegetables, fruits, nuts, red/processed meat, EPA + DHA, PUFA, sodium, and alcohol, and lower in sugar-sweetened beverages (t-test, *p* < 0.001). **(B)** Compared with single group, participants scored higher on sugar-sweetened beverages, nuts, red/processed meat, EPA + DHA, PUFA, and alcohol, and lower on fruit (t-test, *p* < 0.001). Error line representation SD. SD, Standard deviation. ***, *p* < 0.001

There were significant differences in mean AHEI-2010 scores according to education level and marital status. The percentage of all participants with a high school education or above was 68.4%, and the married percentage is 90%. In terms of AHEI-2010 total score, participants above high school (60.17 ± 5.86) were significantly higher than those below high school (54.84 ± 6.13), Fig. 2A, meanwhile, married participants (58.73 ± 6.29) were significantly higher than unmarried participants (56.58 ± 7.25), Fig. 2B. In terms of AHEI-2010 single item score, participants in high school and above scored higher in vegetables, fruits, nuts, red/processed meat, EPA + DHA, PUFA, sodium, and alcohol, and lower in sugar-sweetened beverages, Fig. 3A. Married participants scored higher on sugar-sweetened beverages, nuts, red/processed meat, EPA + DHA, PUFA, and alcohol, and lower on fruit, Fig. 3B. The detailed table is shown in S1-2.

### Correlation of stroke risk

Multivariate logistic regression analysis for stroke risk, Table 4. After adjustment for age, sex, and stroke risk factors, results showed that education level, overweight/obesity, physical inactivity, family history of stroke, and AHEI-2010 total score were independent associated with stroke risk. The results suggest that high education level and marital status are associated with low-risk of stroke. Dyslipidemia, overweight/obesity, and family history of stroke are associated with high-risk of stroke. The detailed table is shown in S3.

**Table 4:** Univariate and multivariate logistic regression analysis of factors associated with stroke risk

Variables	Univariate analysis		Multivariate analysis			
			Model 1^a^		Model 2^b^	
	β coefficient (95% CI)	*p*-value	β coefficient (95% CI)	*p*-value	β coefficient (95% CI)	*p*-value
High School and above	0.098 (0.052–0.182)	<0.001	0.096 (0.051–0.178)	<0.001	0.088 (0.047–0.165)	<0.001
Dyslipidemia	3.397 (2.264–5.097)	<0.001	3.556 (2.358–5.362)	<0.001	4.124 (2.696–6.310)	<0.001
Over weight or obesity	2.861 (1.840–4.448)	<0.001	2.938 (1.881–4.587)	<0.001	3.243 (2.051–5.127)	<0.001
Sufficient physical activity	2.203 (1.566−3.100)	<0.001	2.266 (1.606–3.196)	<0.001	2.470 (1.734–3.519)	<0.001
Family history of stroke	2.797 (1.945–4.022)	<0.001	3.006 (2.069–4.366)	<0.001	3.250 (2.214–4.770)	<0.001
AHEI−2010 Total	0.847 (0.816–0.879)	<0.001	0.847 (0.816–0.879)	<0.001	0.843 (0.812–0.875)	<0.001

^a^Adjusted for sex and age; ^b^Adjusted for sex, age and stroke related risk factors: hypertension, hyperlipidemia, diabetes mellitus, atrial fibrillation or valvular heart disease, current smoking, over weight or obesity, sufficient physical activity, family history of stroke

### Correlation of diet quality

Multivariate logistic regression analysis for quantiles of AHEI-2010, Table 5. After adjustment for age, sex, and stroke risk factors, the results showed that education level, marital status, BMI and AHEI-2010 scores were independent and correlated. The results indicate that high education level and marital status are associated with high AHEI-2010 scores, while high BMI is associated with low AHEI-2010 scores.

**Table 5:** Univariate and multivariate logistic regression analysis of factors associated with AHEI−2010.

	Q1 (n = 153)	Q2 (n = 154)		Q3 (n = 153)		Q4 (n = 154)		Q5 (n = 154)	
	ref.	OR (95% CI)	*p*-value	OR (95% CI)	*p*-value	OR (95% CI)	*p*-value	OR (95% CI)	*p*-value
High School and above									
Unadjusted	1	1.055 (0.624, 1.782)	0.841	0.256 (0.153, 0.431)	<0.001	0.169 (0.099, 0.291)	<0.001	0.086 (0.048, 0.153)	<0.001
Sex and age adjusted	1	1.056 (0.625, 1.786)	0.838	0.258 (0.153, 0.434)	<0.001	0.170 (0.099, 0.291)	<0.001	0.086 (0.048, 0.155)	<0.001
Multivariate adjusted^a^	1	1.058 (0.622, 1.800)	0.835	0.259 (0.153, 0.439)	<0.001	0.168 (0.097, 0.289)	<0.001	0.084 (0.047, 0.152)	<0.001
Current Married									
Unadjusted	1	0.463 (0.219, 0.981)	0.044	0.134 (0.056, 0.320)	<0.001	0.245 (0.119, 0.503)	<0.001	0.137 (0.062, 0.303)	<0.001
Sex and age adjusted	1	0.468 (0.221, 0.993)	0.048	0.133 (0.056, 0.318)	<0.001	0.245 (0.119, 0.505)	<0.001	0.138 (0.063, 0.306)	<0.001
Multivariate adjusted^a^	1	0.467 (0.218, 1.001)	0.050	0.134 (0.056, 0.324)	<0.001	0.246 (0.119, 0.510)	<0.001	0.132 (0.059, 0.294)	<0.001
^1^BMI									
Unadjusted	1	0.879 (0.661, 1.171)	0.379	0.688 (0.512, 1.025)	0.063	0.757 (0.558, 1.026)	0.073	0.826 (0.597, 1.143)	0.248
Sex and age adjusted	1	0.871 (0.653, 1.160)	0.345	0.688 (0.511, 1.025)	0.073	0.752 (0.554, 1.020)	0.067	0.825 (0.595, 1.143)	0.247
Multivariate adjusted^a^	1	0.862 (0.645, 1.153)	0.318	0.685 (0.508, 1.023)	0.073	0.744 (0.547, 1.012)	0.059	0.831 (0.598, 1.154)	0.269
Total cholesterol									
Unadjusted	1	0.976 (0.812, 1.173)	0.795	1.022 (0.848, 1.231)	0.821	1.797 (1.653, 1.972)	0.025	1.782 (1.637, 1.960)	0.019
Sex and age adjusted	1	0.979 (0.814, 1.178)	0.825	1.024 (0.850, 1.233)	0.807	1.798 (1.654, 1.974)	0.026	1.785 (1.639, 1.965)	0.021
Multivariate adjusted^a^	1	0.963 (0.799, 1.159)	0.688	1.014 (0.838, 1.228)	0.886	1.789 (1.645, 1.967)	0.022	1.779 (1.632, 1.960)	0.019
^2^HDL									
Unadjusted	1	1.036 (0.617, 1.739)	0.894	1.070 (0.629, 1.822)	0.802	0.979 (0.557, 1.720)	0.940	0.846 (0.466, 1.538)	0.584
Sex and age adjusted	1	1.028 (0.614, 1.722)	0.917	1.065 (0.623, 1.819)	0.819	0.978 (0.557, 1.717)	0.937	0.839 (0.461, 1.528)	0.566
Multivariate adjusted^a^	1	1.032 (0.611, 1.745)	0.905	1.117 (0.646, 1.932)	0.693	1.006 (0.565, 1.789)	0.985	0.816 (0.445, 1.498)	0.512
^3^TG									
Unadjusted	1	0.832 (0.392, 1.765)	0.632	1.584 (0.714, 3.515)	0.258	1.453 (0.657, 3.214)	0.357	1.170 (0.520, 2.635)	0.705
Sex and age adjusted	1	0.838 (0.395, 1.779)	0.645	1.568 (0.706, 3.484)	0.270	1.467 (0.662, 3.248)	0.345	1.124 (0.498, 2.539)	0.778
Multivariate adjusted^a^	1	0.831 (0.388, 1.781)	0.635	1.544 (0.689, 3.462)	0.291	1.427 (0.639, 3.184)	0.386	1.064 (0.467, 2.424)	0.883
^4^FBS									
Unadjusted	1	1.200 (0.773, 1.863)	0.415	1.460 (0.926, 2.302)	0.103	1.165 (0.717, 1.892)	0.537	0.921 (0.530, 1.603)	0.772
Sex and age adjusted	1	1.223 (0.786, 1.902)	0.372	1.461 (0.926, 2.306)	0.103	1.178 (0.725, 1.916)	0.508	0.928 (0.532, 1.618)	0.791
Multivariate adjusted^a^	1	1.237 (0.791, 1.934)	0.351	1.464 (0.926, 2.314)	0.103	1.197 (0.734, 1.952)	0.471	0.920 (0.526, 1.609)	0.770

^a^Adjusted for sex, age and stroke related risk factors: hypertension, hyperlipidemia, diabetes mellitus, atrial fibrillation or valvular heart disease, current smoking, overweight or obesity, sufficient physical activity, family history of stroke; ^1^BMI: Body Mass Index; ^2^HDL: high-density lipoprotein; ^3^TG: Triglyceride; ^4^FBS: Fasting Blood Sugar

## Discussion

Diet is not only a major issue in developed countries. In China, with increasing prosperity, the consumption of meat and eggs has significantly increased in the past 20 years, while the consumption of fruits and vegetables has decreased.This change has been associated with a 26.6% increase in stroke mortality, between 2003 and 2013 [43]. This study recruited a population from the Tianjin community in China and used AHEI-2010 as a measure of dietary quality. The mean AHEI-2010 score of all participants recruited in this study was 58.49. The total score is 110 points. Although AHEI-2010 has no cutoff point, the average obtained is far from optimal. The study concluded that the diet quality of the Tianjin population is flawed and needs to be improved.

This study found that whether in the low-risk group or high-risk group, fruit is the component with the lowest AHEI-2010 score, which means that the fruit intake of the older population in Tianjin is significantly insufficient. Fruits provide the body with essential nutrients such as vitamins, minerals, dietary fiber, plant compounds. It is an important part of a healthy diet. Insufficient fruit intake is associated with increased morbidity and mortality from chronic diseases such as hypertension [44, 45], cardiovascular disease [46], cancer [47] and stroke [48, 49]. Tianjin population needs to further increase fruit intake. Although sodium is not the lowest scoring component, the high-risk group for stroke scored significantly lower in the AHEI-2010 sodium sub item than the low-risk group. The reason for considering high sodium intake is that Tianjin is close to the ocean and belongs to a high-salt area. High sodium intake is associated with hypertension, higher risk of stroke [50, 51] and overall mortality [52]. Furthermore, in clinical trials, low-sodium diets significantly reduced blood pressure [53] and cardiovascular disease risk [54]. A low-salt diet is one of the keys to staying healthy.

For the sociodemographic variables analyzed in our study, education and marital status were also associated with diet quality, with less educated people having lower overall diet quality, possibly due in part to a lack of nutritional knowledge, cooking skills, or the use of prophylaxis. information capacity [55, 56]. A study found that compared with highly educated women, less educated women have less knowledge of diet-disease relationships, less control over household food choices, and lower social support for healthy eating. As a result, less educated women have fewer economic and psychosocial resources to protect them from harsher environments [57–59]. Getting married often brings health benefits, compared to unmarried people, married people have fewer premature all-cause mortality and chronic health conditions [60]. The presence of an intimate partner is often associated with a reduced risk of coronary heart disease, improved outcomes, more effective health behavior changes, and patient management [61].

Few studies have investigated the relationship between dietary patterns and physical health indicators. A better understanding of the biological basis of diet-related health markers could better explain metabolic differences in individuals with and without chronic disease [62]. This study found that AHEI-2010 quantiles were significantly different among some health indicators (BMI, total cholesterol, HDL, TG and FBS). Among them, total cholesterol was an independent risk factor for AHEI-2010. These findings are clinically important because these health markers are susceptible to negative effects from poor diet. The relationship between laboratory markers and diet is complex because changes in markers are often triggered by the cumulative effects of diet and other factors. In clinical practice, individuals are often advised to make dietary changes, and adherence to healthy eating patterns can lead to improvements in anthropometric and laboratory parameters (i.e., blood glucose, lipids, and homocystrine).

Diet is a complex exposure variable, there are many factors that affect diet, which in turn affects the development of disease. The importance of nutrition in stroke risk is far more important than most physcians suppose. This study found that diet quality was independently and significantly associated with stroke risk. This is consistent with previous research results. Previous studies have found a negative correlation between higher AHEI-2010 scores and stroke risk [40]. A meta-analysis of 20 prospective cohort study showed that, compared to the minimum intake of fruits and vegetables, the lowest intake group had a 21% reduction in stroke risk(95%CI 16-25%) [63]. Another meta-analysis of carbohydrate quality found that the intake of carbohydrates with a lower glycemic index (such as whole grains and dietary fiber) was associated with a 33% reduction in stroke mortality and a 26% reduction in total stroke incidence rate, compared with carbohydrates with a higher glycemic index (refined grains) [64]. Little is known about the impact of diet on stroke subtypes. Chen et al. reported in an network meta-analysis that a Mediterranean diet is associated with a reduction in ischemic and hemorrhagic stroke [65]. The relative risk for ischemic stroke was 0.86, 95%CI 0.81–0.91; for hemorrhagic stroke it was 0.83, 95%CI 0.74–0.93. Therefore, adhering to a healthy dietary pattern and improving dietary quality is crucial for preventing the occurrence of stroke.

## Limitation

This study has limitations. First, this study was a cross-sectional design, and future prospective studies are needed to investigate the causal relationship between diet quality and stroke. Second, we cannot rule out that those participants who were self-aware of their own health might provide healthier lifestyles and thus better adherence to high-quality dietary patterns. Third, to further improve the reliability and generalizability of the study, it is necessary to expand the sample size to a larger scale. In addition, care should be taken in sample selection to consider the diversity across different geographic locations to ensure validity and generalizability of the results.

## Conclusion

In addition to food intake, high education level, married status, blood lipid and blood glucose levels are closely related to the dietary quality of older stroke high-risk groups. The diet quality of the 60–80 year-old community population in Tianjin is far from optimal. Lower diet quality was independently associated with higher stroke risk. There is room for significant improvement in the composition of dietary patterns. It is necessary to strengthen the publicity and education of stroke-related knowledge among community residents and community doctors. Additional steps are needed to impact populations with low levels of education, being single and at high risk of stroke. If so, improvements in diet quality may contribute to the prevention of non-communicable chronic diseases.

### Electronic supplementary material

Below is the link to the electronic supplementary material.

Supplementary Material 1

## Data Availability

The data that support the findings of this study are available from the corresponding author upon reasonable request.
