# Supplementary material for: Factors related to dietary quality among older stroke high-risk population in Tianjin community, China: a multicenter study
Source: BMC Geriatr. 2023 Aug 22;23:508. doi: 10.1186/s12877-023-04211-7 (PMC10463312; doi:10.1186/s12877-023-04211-7)
Supplement: Supplementary file 1 — Supplementary Material 1 [file 12877_2023_4211_MOESM1_ESM.docx]

| Supplementary table 1 AHEI-2010 total score and each component score of different educational levels | | | |
| --- | --- | --- | --- |
| AHEI-2010 | High School and above  (n=731) | Below high school  (n=337) | *p*-value |
| Vegetables | 6.75 (1.03) | 6.16 (1.19) | ＜0.001 |
| Fruit | 1.90 (0.42) | 1.67 (0.19) | ＜0.001 |
| Whole grains | 6.18 (1.35) | 6.09 (1.38) | 0.315 |
| Sugar-sweetened beverages and fruit juice | 5.49 (0.71) | 5.61 (0.60) | 0.007 |
| Nuts and legumes | 5.52 (0.22) | 4.70 (0.43) | ＜0.001 |
| Red/processed meat | 4.81 (0.87) | 4.21 (1.03) | ＜0.001 |
| trans Fat | 7.26 (0.90) | 7.22 (0.70) | 0.471 |
| Long-chain (ω-3) fats (EPA + DHA) | 5.06 (0.04) | 4.22 (0.37) | ＜0.001 |
| Polyunsaturated fatty acids | 7.25 (1.10) | 6.83 (1.58) | ＜0.001 |
| Sodium | 6.53 (1.42) | 5.40 (1.75) | ＜0.001 |
| Alcohol | 3.55 (0.16) | 2.97 (0.68) | ＜0.001 |
| Total | 60.17 (5.86) | 54.84 (6.13) | ＜0.001 |

| Supplementary table 2 AHEI-2010 total score and each component score of different marital status | | | |
| --- | --- | --- | --- |
| AHEI-2010 | Current Married  (n=961) | Single  (n=107) | *p*-value |
| Vegetables | 6.59 (1.08) | 6.42 (1.09) | 0.123 |
| Fruit | 1.80 (0.31) | 2.11 (0.70) | ＜0.001 |
| Whole grains | 6.16 (1.35) | 6.02 (1.42) | 0.312 |
| Sugar-sweetened beverages and fruit juice | 5.54 (0.67) | 5.37 (0.79) | 0.015 |
| Nuts and legumes | 5.30 (0.31) | 4.89 (0.40) | ＜0.001 |
| Red/processed meat | 4.65 (0.94) | 4.45 (0.95) | 0.037 |
| trans Fat | 7.25 (0.82) | 7.20 (0.97) | 0.557 |
| Long-chain (ω-3) fats (EPA + DHA) | 4.82 (0.19) | 4.65 (0.10) | ＜0.001 |
| Polyunsaturated fatty acids | 7.14 (1.23) | 6.85 (1.56) | 0.025 |
| Sodium | 6.21 (1.56) | 5.96 (1.66) | 0.119 |
| Alcohol | 3.43 (0.10) | 2.79 (0.32) | ＜0.001 |
| Total | 58.73 (6.29) | 56.58 (7.25) | ＜0.001 |

| Supplementary table 3. Univariate and multivariate logistic regression analysis of factors associated with stroke risk. | | | | | | |
| --- | --- | --- | --- | --- | --- | --- |
| Variables | Univariate analysis | | Multivariate analysis | | | |
|  |  |  | Model 1^a^ | | Model 2^b^ | |
|  | β coefficient (95% CI) | *p*-value | β coefficient (95% CI) | *p*-value | β coefficient (95% CI) | *p*-value |
| Educaton Level | 0.098 (0.052-0.182) | ＜0.001 | 0.096 (0.051-0.178) | ＜0.001 | 0.088 (0.047-0.165) | ＜0.001 |
| Dyslipidemia | 3.397 (2.264-5.097) | ＜0.001 | 3.556 (2.358-5.362) | ＜0.001 | 4.124 (2.696-6.310) | ＜0.001 |
| Over weight or obesity | 2.861 (1.840-4.448) | ＜0.001 | 2.938 (1.881-4.587) | ＜0.001 | 3.243 (2.051-5.127) | ＜0.001 |
| Sufficient physical activity | 2.203 (1.566-3.100) | ＜0.001 | 2.266 (1.606-3.196) | ＜0.001 | 2.470 (1.734-3.519) | ＜0.001 |
| Family history of stroke | 2.797 (1.945-4.022) | ＜0.001 | 3.006 (2.069-4.366) | ＜0.001 | 3.250 (2.214-4.770) | ＜0.001 |
| AHEI-2010 Total | 0.847 (0.816-0.879) | ＜0.001 | 0.847 (0.816-0.879) | ＜0.001 | 0.843 (0.812-0.875) | ＜0.001 |
| BMI | 1.001 (0.925-1.083) | 0.982 | 0.999 (0.930-1.074) | 0.986 | 1.004 (0.923-1.093) | 0.926 |
| Total cholesterol | 1.081 (0.955-1.225) | 0.219 | 1.088 (0.960-1.233) | 0.185 | 1.073 (0.946-1.218) | 0.274 |
| FBS | 1.028 (0.861-1.226) | 0.762 | 1.033 (0.874-1.221) | 0.704 | 1.014 (0.842-1.221) | 0.884 |
| Homocysteine | 1.004 (0.985-1.023) | 0.660 | 1.005 (0.987-1.025) | 0.577 | 1.005 (0.986-1.024) | 0.614 |
| Female | - | - | 1.359 (0.970-1.903) | 0.074 | 1.049 (0.729-1.509) | 0.797 |
| Age | - | - | 1.010 (0.991-1.028) | 0.302 | 1.015 (0.996-1.034) | 0.114 |
| Current smoking | - | - | - | - | 2.485 (1.520-4.064) | ＜0.001 |

^a^Adjusted for sex and age; ^b^Adjusted for sex, age and stroke related risk factors: hypertension, hyperlipidemia, diabetes mellitus, atrial fibrillation or valvular heart disease, current smoking, over weight or obesity, sufficient physical activity, family history of stroke.
